# Supplementary material for: Mapping the evolution of fertility support policies in China: A content and instrumental analysis
Source: PLoS One. 2025 Oct 9;20(10):e0332137. doi: 10.1371/journal.pone.0332137 (PMC12510515; doi:10.1371/journal.pone.0332137)
Supplement: S1 Appendix — (ZIP) [file pone.0332137.s001.zip › S1 Appendix. 226 original policy documents/192-国家发展改革委、市场监管总局关于新时代服务业高质量发展的指导意见(FBM-CLI-4-336795).doc]

国家发展改革委、市场监管总局关于新时代服务业高质量发展的指导意见

**发布部门：** [国家发展和改革委员会(含原国家发展计划委员会、原国家计划委员会)](https://www.pkulaw.com/law/chl?Aggs.IssueDepartment=60301) [国家市场监督管理总局](https://www.pkulaw.com/law/chl?Aggs.IssueDepartment=60426) [机构沿革](javascript:void(0))

**发文字号：**发改产业〔2019〕1602号

**发布日期：**2019.10.02

**实施日期：**2019.10.02

**时效性：** [现行有效](https://www.pkulaw.com/law/chl?Aggs.TimelinessDic=01)

**效力级别：** [部门规范性文件](https://www.pkulaw.com/law/chl?Aggs.EffectivenessDic=XE0302)

**法规类别：** [服务](https://www.pkulaw.com/law/chl?Aggs.Category=07102) [营商环境优化](https://dobusiness.pkulaw.com/)

国家发展改革委、市场监管总局关于新时代服务业高质量发展的指导意见
（发改产业〔2019〕1602号）

国务院有关部门，各省、自治区、直辖市及计划单列市、新疆生产建设兵团发展改革委、市场监管局：
　　为深入贯彻党的十九大精神，落实《中共中央、国务院关于推动高质量发展的意见》要求，促进我国服务业高质量发展，国家发展改革委、市场监管总局制定《关于新时代服务业高质量发展的指导意见》。指导意见突出宏观统筹，围绕制约服务业高质量发展的薄弱环节和共性问题部署任务，分行业高质量发展由行业主管部门部署实施。
　　**一、**总体思路
　　（一）指导思想。
　　以习近平新时代中国特色社会主义思想为指导，全面贯彻党的十九大和十九届二中、三中全会精神，统筹推进“五位一体”总体布局，协调推进“四个全面”战略布局，坚定践行新发展理念，深化服务业供给侧结构性改革，支持传统服务行业改造升级，大力培育服务业新产业、新业态、新模式，加快发展现代服务业，着力提高服务效率和服务品质，持续推进服务领域改革开放，努力构建优质高效、布局优化、竞争力强的服务产业新体系，不断满足产业转型升级需求和人民美好生活需要，为实现经济高质量发展提供重要支撑。
　　（二）主要原则。
　　以人为本，优化供给。坚持以人民为中心的发展思想，更多更好满足多层次多样化服务需求，不断增强人民的获得感、幸福感、安全感。优先补足基本公共服务短板，着力增强非基本公共服务市场化供给能力，实现服务付费可得、价格合理、优质安全，以高质量的服务供给催生创造新的服务需求。
　　市场导向，品牌引领。顺应产业转型升级新趋势，充分发挥市场配置资源的决定性作用，更好发挥政府作用，在公平竞争中提升服务业竞争力。坚持质量至上、标准规范，树立服务品牌意识，发挥品牌对服务业高质量发展的引领带动作用，着力塑造中国服务品牌新形象。
　　创新驱动，跨界融合。贯彻创新驱动发展战略，推动服务技术、理念、业态和模式创新，增强服务经济发展新动能。促进服务业与农业、制造业及服务业不同领域间的融合发展，形成有利于提升中国制造核心竞争力的服务能力和服务模式，发挥中国服务与中国制造组合效应。
　　深化改革，扩大开放。深化服务领域改革，破除制约服务业高质量发展的体制机制障碍，优化政策体系和发展环境，最大限度激发发展活力和潜力。推动服务业在更大范围、更宽领域、更深层次扩大开放，深度参与国际分工合作，鼓励服务业企业在全球范围内配置资源、开拓市场。
　　（三）总体目标。
　　到2025年，服务业增加值规模不断扩大，占GDP比重稳步提升，吸纳就业能力持续加强。服务业标准化、规模化、品牌化、网络化和智能化水平显著提升，生产性服务业效率和专业化水平显著提高，生活性服务业满足人民消费新需求能力显著增强，现代服务业和先进制造业深度融合，公共服务领域改革不断深入。服务业发展环境进一步改善，对外开放领域和范围进一步扩大，支撑经济发展、民生改善、社会进步的功能进一步增强，功能突出、错位发展、网络健全的服务业高质量发展新格局初步形成。

　　**二、**重点任务
　　（一）推动服务创新。
　　加强技术创新和应用，打造一批面向服务领域的关键共性技术平台，推动人工智能、云计算、大数据等新一代信息技术在服务领域深度应用，提升服务业数字化、智能化发展水平，引导传统服务业企业改造升级，增强个性化、多样化、柔性化服务能力。鼓励业态和模式创新，推动智慧物流、服务外包、医养结合、远程医疗、远程教育等新业态加快发展，引导平台经济、共享经济、体验经济等新模式有序发展，鼓励更多社会主体围绕服务业高质量发展开展创新创业创造。推动数据流动和利用的监管立法，健全知识产权侵权惩罚性赔偿制度，建设国家知识产权服务业集聚发展区。
　　（二）深化产业融合。
　　加快发展农村服务业，引导农业生产向生产、服务一体化转型，探索建立农业社会化服务综合平台，推动线上线下有机结合；支持利用农村自然生态、历史遗产、地域人文、乡村美食等资源，发展乡村旅游、健康养老、科普教育、文化创意、农村电商等业态，推动农业“接二连三”。打造工业互联网平台，推动制造业龙头企业技术研发、工业设计、采购分销、生产控制、营运管理、售后服务等环节向专业化、高端化跃升；大力发展服务型制造，鼓励有条件的制造业企业向一体化服务总集成总承包商转变；开展先进制造业与现代服务业融合发展试点。以大型服务平台为基础，以大数据和信息技术为支撑，推动生产、服务、消费深度融合；引导各地服务业集聚区升级发展，丰富服务功能，提升产业能级；推进港口、产业、城市融合发展；深入开展服务业综合改革试点。
　　（三）拓展服务消费。
　　补齐服务消费短板，激活幸福产业潜在服务消费需求，全面放开养老服务市场，在扩大试点基础上全面建立长期护理保险制度；简化社会办医审批流程，鼓励有实力的社会机构提供以先进医疗技术为特色的医疗服务；加快建立远程医疗服务体系，推动优质资源下沉扩容；支持社会力量兴办托育服务机构。打造中高端服务消费载体，吸引健康体检、整形美容等高端服务消费回流。推动信息服务消费升级、步行街改造提升，支持有条件的地方建设新兴消费体验中心，开展多样化消费体验活动。鼓励企业围绕汽车、家电等产品更新换代和消费升级，完善维修售后等配套服务体系。着力挖掘农村电子商务和旅游消费潜力，优化农村消费市场环境。完善消费者保护机制，打造一批放心企业、放心网站、放心商圈和放心景区。
　　（四）优化空间布局。
　　围绕京津冀协同发展、粤港澳大湾区建设、推进海南全面深化改革开放、长江三角洲区域一体化发展等国家战略，建设国际型、国家级的现代服务经济中心，形成服务业高质量发展新高地。推动城市群和都市圈公共服务均等化和要素市场一体化，构建城市群和都市圈服务网络，促进服务业联动发展和协同创新，形成区域服务业发展新枢纽。强化中小城市服务功能，打造一批服务业特色小镇，形成服务周边、带动农村的新支点。完善海洋服务基础设施，积极发展海洋物流、海洋旅游、海洋信息服务、海洋工程咨询、涉海金融、涉海商务等，构建具有国际竞争力的海洋服务体系。
　　（五）提升就业能力。
　　大力发展人力资源服务业，培育专业化、国际化人力资源服务机构，加快人力资源服务产业园建设，鼓励发展招聘、人力资源服务外包和管理咨询、高级人才寻访等业态。支持企业和社会力量兴办职业教育，鼓励发展股份制、混合所有制等多元化职业教育集团（联盟），完善职业教育和培训体系。鼓励普通高等学校、职业院校增设服务业相关专业，对接线上线下教育资源，推动开展产教融合型城市和企业建设试点。围绕家政服务、养老服务、托育服务、健康养生、医疗护理等民生领域服务需求，提升从业人员职业技能，增强服务供需对接能力。
　　（六）建设服务标准。
　　瞄准国际标准，推动国际国内服务标准接轨，鼓励社会团体和企业制定高于国家标准或行业标准的团体标准、企业标准。完善商贸旅游、社区服务、物业服务、健康服务、养老服务、休闲娱乐、教育培训、体育健身、家政服务、保安服务等传统服务领域标准，加快电子商务、供应链管理、节能环保、知识产权服务、商务服务、检测认证服务、婴幼儿托育服务、信息技术服务等新兴服务领域标准研制。开展服务标准、服务认证示范，推动企业服务标准自我声明公开和监督制度全面实施。
　　（七）塑造服务品牌。
　　支持行业协会、第三方机构和地方政府开展服务品牌培育和塑造工作，树立行业标杆和服务典范，选择产业基础良好、市场化程度较高的行业，率先组织培育一批具有国际竞争力的中国服务品牌和具有地方特色的区域服务品牌。研究建立服务品牌培育和评价标准体系，引导服务业企业树立品牌意识，运用品牌培育的标准，健全品牌营运管理体系。加强服务品牌保护力度，依法依规查处侵权假冒服务品牌行为。开展中国服务品牌宣传、推广活动，以“一带一路”建设为重点，推动中国服务走出去。
　　（八）改进公共服务。
　　紧密围绕城乡居民优质便利生活需求，统筹规划公园绿地、无障碍通道、公共交通、停车场地、社区卫生中心、村卫生室、村级综合文化服务中心等基础设施建设，合理布局社区养老、托育中心、便利店、洗衣房、售后维修、物流快递等便民服务设施，提升各类公共文化、体育场馆免费或低收费开放服务水平。制定完整社区建设标准，明确社区各类服务设施配置标准和建设要求。推进政务服务“一网通办”、现场办理“最多跑一次”，提高政府服务群众、服务企业水平和能力。加快政务信息系统整合，建立全国统一、多级互联的数据共享交换平台体系。在保障信息安全前提下，建立健全税务、市场监管、社保、海关、医疗机构等领域的信息查询系统，提高标准化、便利化、规范化水平。建立政务服务“好差评”制度，提高柔性化治理、精细化服务水平。
　　（九）健全质量监管。
　　推动服务业企业采用先进质量管理模式方法，公开服务质量信息，实施服务质量承诺，开展第三方认证。制定服务质量监测技术指南等规范，加快构建模型统一、方法一致、结果可比的服务质量监测体系。加强服务质量监测评价技术机构布局建设，服务质量监测评价能力和范围基本覆盖到主要服务行业和公共服务领域，定期通报监测结果，督促引导社会各方提高服务质量水平。加快服务质量监管立法，建立健全服务质量监管协同处置机制，及时依法调查处理重大服务质量安全事件，不断完善服务质量治理体系。
　　（十）扩大对外开放。
　　稳步扩大金融业开放，加快电信、教育、医疗、文化等领域开放进程，赋予自贸试验区更大改革自主权。积极引进全球优质服务资源，增强服务业领域国际交流与合作，以“一带一路”建设为重点，引导有条件的企业在全球范围配置资源、拓展市场，推动服务业和制造业协同走出去。大力发展服务贸易，巩固提升旅游、建筑、运输等传统服务贸易，拓展中医药等中国特色服务贸易，培育文化创意、数字服务、信息通讯、现代金融、广告服务等新兴服务贸易，扩大研发设计、节能环保、质量管理等高技术服务进出口。

　　**三、**政策保障
　　（一）优化营商环境。
　　深化服务业“放管服”改革，进一步压缩企业开办时间和服务商标注册周期。深化企业简易注销改革，试点进一步压缩公告时间和拓展适用范围。取消企业名称预先核准，开展扩大企业名称自主申报改革试点。推动“非禁即入”普遍落实，全面实施[市场准入负面清单](https://www.pkulaw.com/chl/d8135b05437494cebdfb.html)制度。制定加快放宽服务业市场准入的意见。坚决查处垄断协议、滥用市场支配地位和滥用行政权力排除限制竞争的行为。对服务业新产业、新业态、新模式，坚持包容审慎监管原则，在质量监控、消费维权、税收征管等方面实施线上线下一体化管理。推进服务市场信用体系建设，建立市场主体信用记录，健全对失信主体的惩戒机制。探索建立涉及民生安全的重点服务领域从业人员守信联合激励和失信联合惩戒制度，完善服务消费领域信用信息共享共用机制。加强服务环境综合治理，强化服务业价格监管，及时查处消费侵权等问题。
　　（二）加大融资支持。
　　进一步完善有关金融政策，引导金融机构在风险可控、商业可持续的前提下创新机制和产品，按照市场化、商业化原则拓展企业融资渠道。鼓励金融机构积极运用互联网技术，打通企业融资“最后一公里”，更好地满足中小企业融资需求。探索通过新技术、新模式，进一步优化中小企业银行账户服务。发展动产融资，依托现有交易市场，合规开展轻资产交易，缓解中小服务业企业融资难题。引导创业投资加大对中小服务业企业的融资支持，支持符合条件的技术先进型服务业企业上市融资，支持科技型企业利用资本市场做大做强。
　　（三）强化人才支撑。
　　鼓励服务业从业人员参加职业技能鉴定（或职业技能等级认定、专项职业能力考核），对通过初次职业技能鉴定并取得职业资格证书（或职业技能登记证书、专项职业能力证书）的，按规定给予一次性职业技能鉴定补贴。进一步畅通非公经济组织人员和自由职业者职称申报渠道。实施更加开放的人才引进政策，加大对海外高端服务业人才的引进力度，改革完善人才培养、使用、评价机制。运用股权激励递延纳税等政策，鼓励服务业企业采用股权激励等中长期激励方式引留人才。完善灵活就业人员社会保险政策。
　　（四）保障用地需求。
　　优化土地供应调控机制，保障服务业高质量发展用地需求。适应服务业新产业、新业态、新模式特点，创新用地供给方式。实施“退二进三”“退低进高”，对提高自有工业用地容积率用于自营生产性服务业的工业企业，依法按新用途办理相关手续。加强历史建筑的活化利用，有效发挥历史建筑服务功能。
　　（五）落实财税和价格政策。
　　落实支持服务业发展的税收优惠政策，做好政策宣传和纳税辅导，确保企业充分享受政策红利。加大政府购买服务力度，扩大购买范围，优化政府购买服务指导性目录，加强购买服务绩效评价。降低一般工商业电价，全面落实工商用电同价政策，推动地方落实国家鼓励类服务业用水与工业同价；在实行峰谷电价的地区，有条件的地方可以开展商业用户选择执行行业平均电价或峰谷分时电价试点。落实社区养老服务机构税费减免、资金支持、水电气热价格优惠等扶持政策。
　　（六）建立健全统计制度。
　　健全服务业统计调查制度，建立健全生产性、生活性服务业统计分类，完善统计分类标准和指标体系，提高统计数据及时性和精准度。逐步建立生产性、生活性服务业统计信息定期发布制度，建立健全服务业重点领域统计信息在部门间的共享机制，逐步形成年度、季度信息发布机制。
　　各地区、各有关部门要强化主体责任，形成工作合力，认真落实指导意见各项任务要求。各地方要加强组织领导，结合实际抓好贯彻落实，切实推动本地区服务业高质量发展，及时向有关部门报告进展情况。各有关部门要按照职责分工，细化制定配套政策，加强对地方工作的督导，推动指导意见有效落实。要充分发挥服务业发展部际联席会议制度作用，细化实化工作任务和完成时限，适时开展服务业高质量发展评估工作，加强对指导意见实施的督促检查，扎实推动服务业高质量发展取得实效。

国家发展改革委
市场监管总局
2019年10月2日

© 北大法宝：（[www.pkulaw.com](https://www.pkulaw.com/)) 专业提供法律信息、法学知识和法律软件领域各类解决方案。北大法宝为您提供丰富的参考资料，正式引用法规条文时请与标准文本核对。
欢迎查看所有[产品和服务](https://www.pkulaw.com/helps/2.html)。法宝快讯：[如何快速找到您需要的检索结果？](https://www.pkulaw.com/helps/70.html)[法宝](https://www.pkulaw.com/helps/69.html)V6有何新特色？

扫描二维码阅读原文

**原文链接：**<https://www.pkulaw.com/chl/922054bc8ed21ff9bdfb.html>
